# Laparoscopic Repair of Blunt Traumatic Diaphragmatic Hernia

**DOI:** 10.7759/cureus.46017

**Published:** 2023-09-26

**Authors:** Maaz A Yusufi, Muhammad Uneeb, Izza Nazir, Farhan Rashid

**Affiliations:** 1 General Surgery, Shifa International Hospital Islamabad, Islamabad, PAK; 2 General Surgery, Bahria International Hospital, Rawalpindi, PAK

**Keywords:** emergency laparoscopy, minimally invasive surgery, blunt force trauma, traumatic diaphragmatic hernia, diaphragmatic rupture

## Abstract

Traumatic diaphragmatic hernias (TDHs) can occur after both blunt and penetrating injury. Laparotomy and thoracotomy are commonly done for the management of TDHs. Minimally invasive surgery, especially laparoscopic surgery, is being accepted as an effective and safe alternative to open surgical repair even in trauma cases. Laparoscopy also allows for the detection and management of clinically occult TDHs, thereby preventing the complications of missed or delayed diagnosis. Our case highlights the importance of timely intervention with a minimally invasive approach.

A 39-year-old male presented to the emergency room after a road traffic accident. Computed tomography scan confirmed left-sided diaphragmatic rupture with gastric herniation. Laparoscopic repair of the hernia was done. He had an uneventful post-operative period. At the one-year follow-up, he was asymptomatic and was doing well.

TDHs have a variable clinical presentation and radiological findings are not always diagnostic. Such cases can progress to potentially life-threatening complications such as strangulation and perforation of the herniated viscera. Timely diagnosis and management are therefore essential. A minimally invasive approach such as laparoscopy should be used for the management of TDHs in the acute setting where the patient is stable, and resources are available. In this case, once the gastric contents were aspirated via a nasogastric tube in the middle of the night, the immediate need for surgery was converted to an urgent nature, and the patient underwent surgery the next morning in a more controlled setting. In addition, timely intervention can prevent future complications that may occur if the condition is left untreated during the initial admission.

## Introduction

Traumatic diaphragmatic hernias (TDHs) can occur after both blunt and penetrating trauma. The diaphragm is a dome-shaped muscle separating the positive pressure in the abdominal cavity from the negative pressure in the thoracic cavity. This positive pressure gradient between the peritoneal and pleural spaces measures about 7-20 cm of water. When the diaphragm is ruptured, the viscera follow the pressure gradient and herniate from the abdominal cavity into the thoracic cavity [1].

Laparotomy is commonly done for the management of TDHs. Although the first laparoscopic repair of a diaphragmatic injury was done in 1994 for a penetrating injury, the open approach is still commonly used as the standard [2]. However, minimally invasive surgery, especially laparoscopic surgery, is being accepted as an effective and safe alternative to open surgical repair even in trauma cases. Kim et al. reported a case of robotic transthoracic repair of a TDH [3]. Surgical repair is indicated even if the patient is asymptomatic as there is a risk of incarceration and strangulation of the herniated viscera. A minimally invasive approach such as laparoscopy is safe and effective and has the added advantage of being diagnostic as well as therapeutic, in addition to the classic benefits such as decreased post-operative pain and earlier recovery.

This article was previously presented as a poster at the Biennial National Surgical Conference, Surgicon 2022, Society of Surgeons of Pakistan (Lahore chapter), on December 03, 2022.

## Case presentation

A 39-year-old male presented to the emergency room (ER) after his car rolled over and struck an oncoming sports utility vehicle (SUV). He was the driver of the car, while the passenger died on the spot. At presentation, he was tachycardic, with a GCS of 3/15, for which he was intubated in the ER. On examination, he had a 5 cm laceration on his forehead, along with decreased air entry at the base of the left lung.

**Figure 1:**
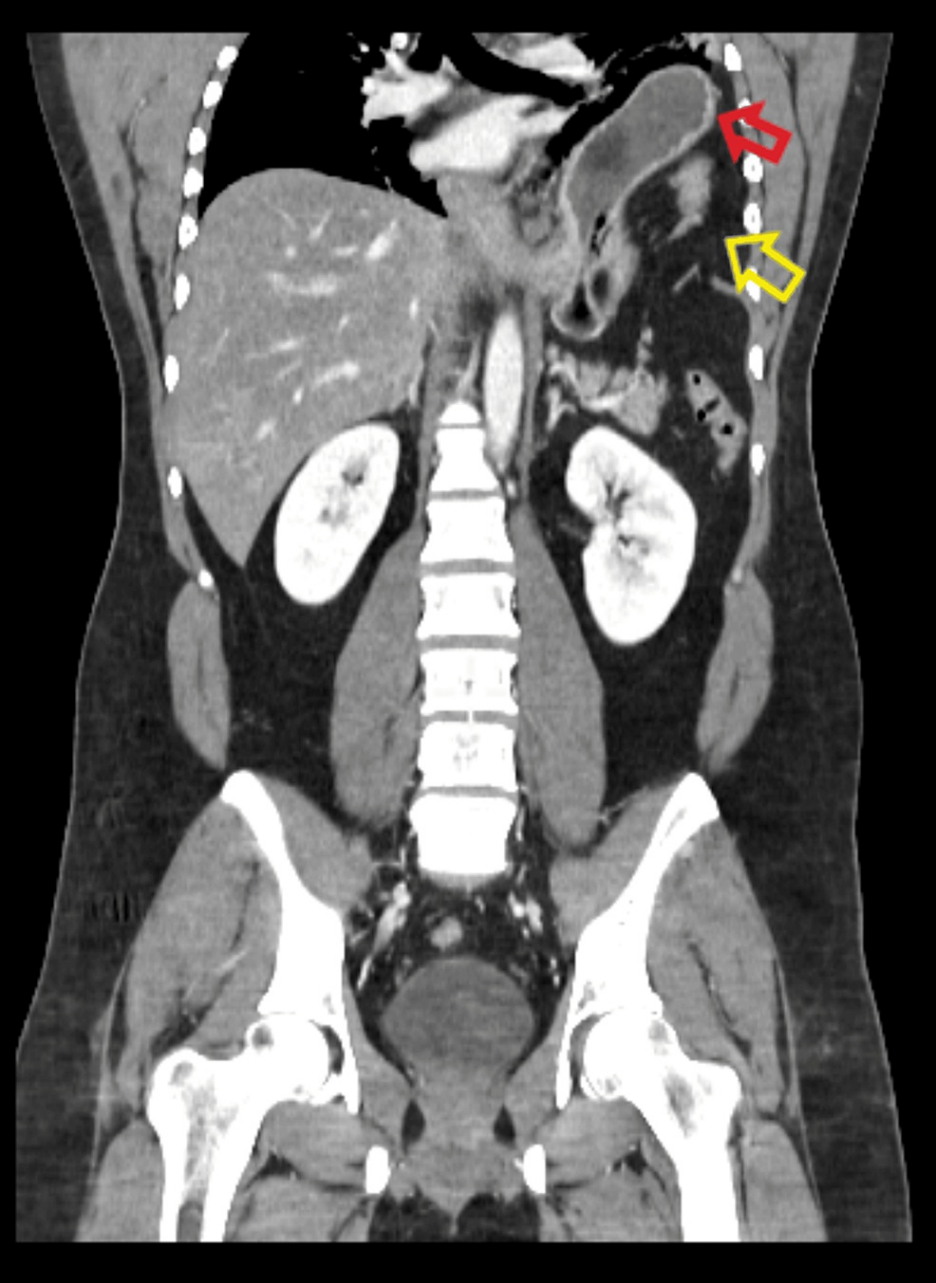
Computed tomography scan done at the initial presentation (coronal view) showing left-sided diaphragmatic hernia, with the stomach (red arrow) and the omentum (yellow arrow) in the left hemithorax

**Figure 2:**
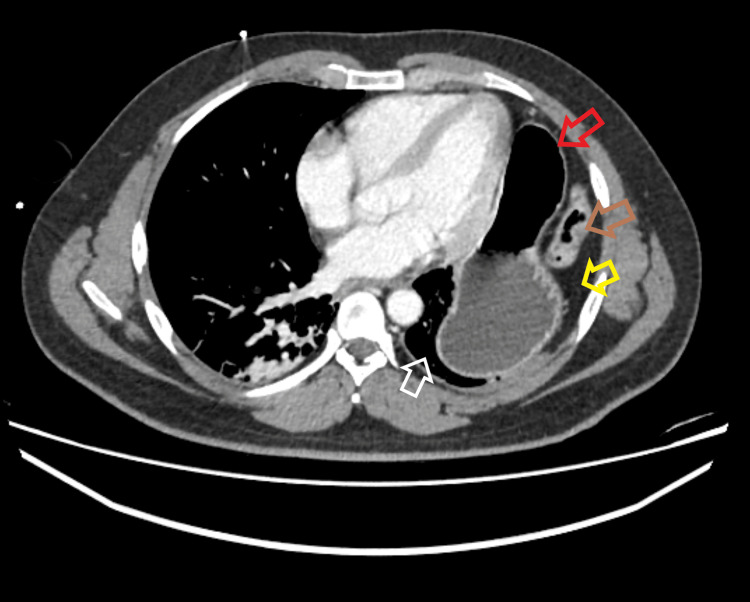
Computed tomography scan done at the initial presentation (axial view) showing the herniated stomach (red arrow), omentum (yellow arrow), and colon (brown arrow), all within the left hemithorax, causing the left lung (white arrow) to be collapsed

After initial resuscitation, a whole body computed tomography (CT) scan was done in the ER according to the trauma protocol, which showed a fracture of the tenth and eleventh ribs on the left side, as well as a large left-sided diaphragmatic hernia through which the stomach, omentum, and colon had herniated (Figures 1, 2). Basilar skull fracture was ruled out on the CT scan. A nasogastric (NG) tube was placed, through which 300 ml of gastric contents were aspirated.

**Figure 3:**
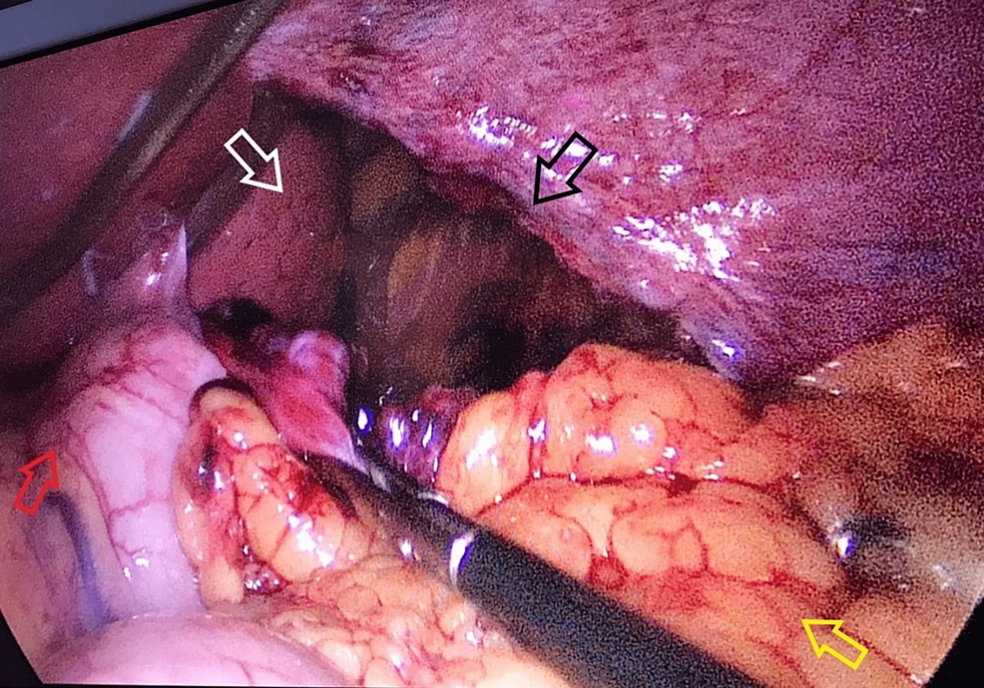
Intra-operative image showing a large tear in the left hemidiaphragm (black arrow), through which the stomach (red arrow) and omentum (yellow arrow) had herniated, and can be seen to be partially reduced. The lung (white arrow) is collapsed

A laparoscopic repair of the TDH was planned, and the patient was taken to the operating room (OR) within a few hours. The open Hasson technique was used to create pneumoperitoneum, and a five-port approach was used. Intra-operative findings confirmed a 10 cm rupture of the medial end of the left dome of the diaphragm through which the stomach and omentum had herniated (Figure 3). The gastroesophageal junction was below the diaphragm, and there was no evidence of visceral perforation or gangrene.

**Figure 4:**
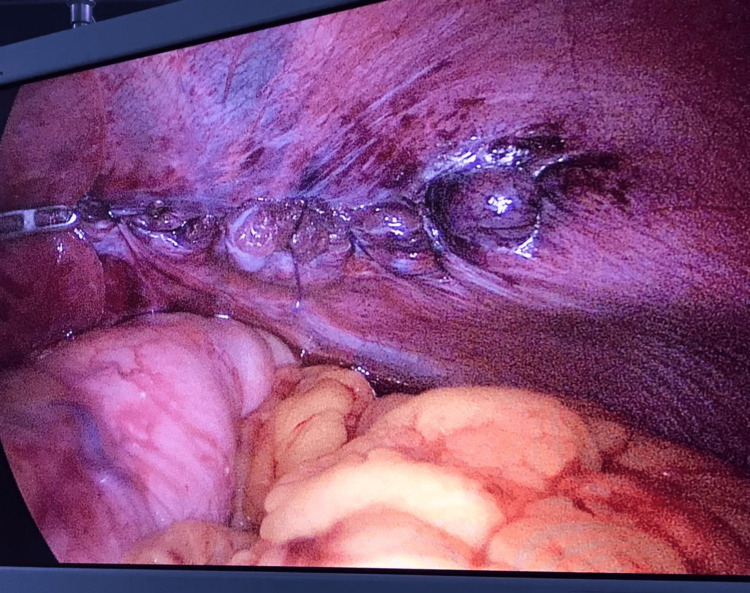
Intra-operative image showing the left hemidiaphragm which has been repaired with continuous sutures after reduction of the herniated contents

The herniated stomach and omentum were reduced into the abdominal cavity. Primary repair of the left dome of the diaphragm was done using 2/0 V-LocTM 90 sutures (Covidien Healthcare, Mansfield, Massachusetts) in a continuous manner, as shown in Figure 4. A chest drain (28 Fr) was placed in the left thoracic cavity under vision, before closure of the diaphragm, after visualizing the inflated lower lobe of the left lung using the Valsalva maneuver. Another drain was placed below the left diaphragm in the left upper quadrant of the abdomen.

**Figure 5:**
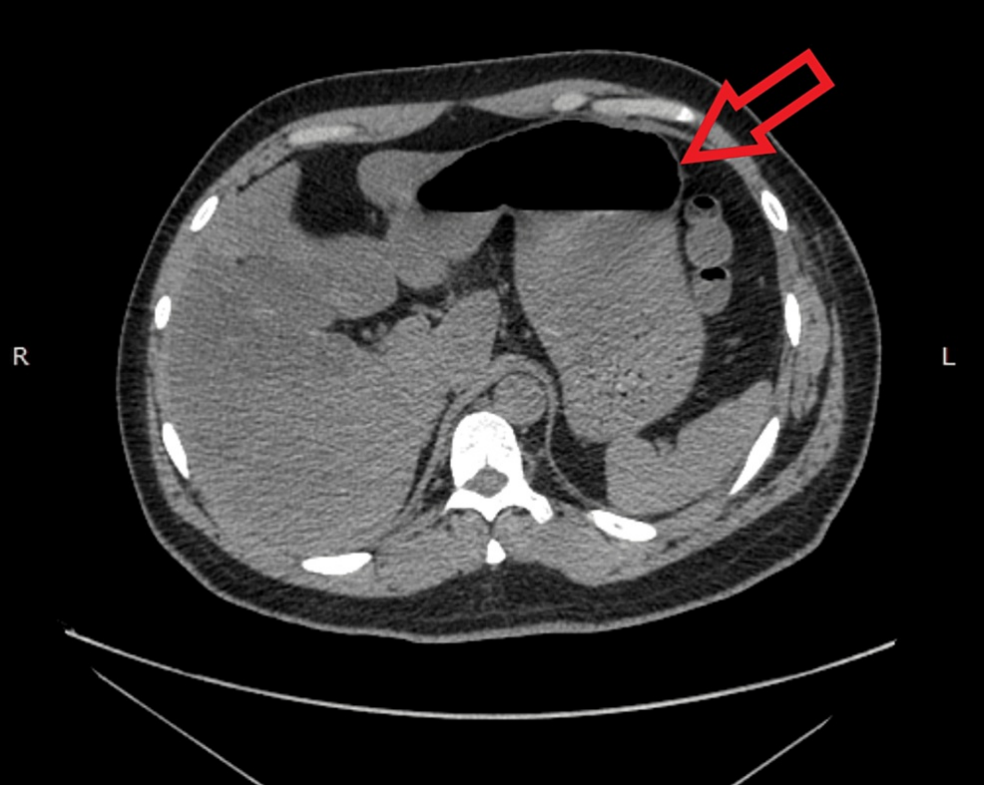
Computed tomography scan done 20 days after the surgery (axial view) showing the stomach (red arrow) in the abdominal cavity, and no evidence of diaphragmatic herniation

He was allowed clear liquids per oral from the first post-operative day, with progression to soft diet from the second post-operative day. His chest and abdominal drains were removed on the fourth post-operative day. He was discharged in stable condition on the eighth post-operative day. On the twentieth post-operative day, he developed gastroenteritis and went to a local hospital. Due to the recent surgery, the emergency physician vigilantly performed a CT scan of the chest to rule out any post-operative complications. It showed that the diaphragmatic repair was intact, without any evidence of herniation (Figure 5). At the one-year follow-up, he was asymptomatic and was doing well.

## Discussion

Traumatic diaphragmatic rupture and herniation are relatively uncommon. However, they are often occult injuries due to silent initial presentation and non-specific imaging findings. As a result, they often remain undiagnosed at the time of presentation. This can have long-term complications and is associated with significant morbidity and mortality. If left untreated, the mortality of TDHs can reach as high as 66% due to the incarceration and subsequent strangulation of the herniated viscera, as well as injury to the thoracic viscera. Therefore, the prompt identification of diaphragmatic hernias is essential and is an indication of immediate surgical intervention [4].

The presentation of TDHs is highly variable. There are often both respiratory and abdominal symptoms [5]. These depend on several factors, including the type and severity of trauma, the side with greater injury, the herniated viscera, and the associated injuries. In most cases, there is an increase in the pressure gradient between the abdominal and thoracic compartments, with rupture at the weakest points of the diaphragm, which are usually the embryonic melting points. In some cases, the hernia may be completely asymptomatic at the initial presentation, or even for several years thereafter, before progressing only to vague symptoms, until finally incarceration and strangulation create a surgical emergency [6].

TDHs can be classified based on the time of diagnosis into three categories. Type 1 hernia is when the diagnosis is made immediately following trauma. In type 2 hernia, the diagnosis is made within the recovery period. Finally, type 3 hernia is when the diagnosis is delayed until the patient presents with ischemia or perforation of the herniated viscera [7].

Various imaging modalities can be used for the diagnosis of blunt traumatic diaphragmatic rupture with or without herniation. These include chest X-ray, CT scan, and barium studies, for the evaluation of the diaphragmatic defect, including its size, location, and contents [5]. However, CT scan is the reference standard. A number of CT signs have been described for TDHs. Although the accuracy of diagnosis increases when more than one sign is present, even a single sign in the context of blunt trauma should be considered suspicious for TDHs. Magnetic resonance imaging can be more useful for diagnosis; however, it is usually not feasible in the case of trauma patients, for whom ultrasound may be a more viable option [7].

There are at least 19 documented CT signs, which can be classified into direct signs, indirect signs, and signs of uncertain or controversial origin. The direct signs include segmental diaphragmatic defect, Dangling Diaphragm sign, and absent diaphragm. The indirect signs can be further divided into those related to herniation, and those related to a loss of border between the thorax and abdomen. The indirect signs related to herniation include herniation through a defect, Collar sign (hourglass constriction sign or mushroom sign, in case of hepatic hernia), hump sign, band sign, dependent viscera, sinus cutoff sign, abdominal content peripheral to the diaphragm or lung, and elevated abdominal organs. The indirect signs related to loss of border between thorax and abdomen include abdominal fluid abutting a thoracic structure, abdominal viscera abutting thoracic fluid or a thoracic organ, pneumothorax/pneumoperitoneum, and hemothorax/hemoperitoneum. Finally, signs of uncertain or controversial origin include thickening of the diaphragm (curled diaphragm sign), diaphragmatic or peri-diaphragmatic contrast medium extravasation, hypoenhanced or hypoattenuated diaphragm, and fractured rib (with presumed laceration of diaphragm by rib trajectory) [8].

The diaphragm tends to get ruptured at either the central tendon or the boundary between the central tendon and the muscular parts of the diaphragm. The left side is more commonly injured, with a reported 69 to 90% of TDH occurring on the left side. In contrast, the right side is only affected in about 9-24% of cases. The right-sided TDH tends to be more frequently undiagnosed, due to the low diagnostic accuracy of imaging modalities for the right-sided TDH than for the left-sided TDH [4]. A bilateral TDH occurs in a minority of cases. There are several reasons for the increased incidence of left-sided TDH. The right hemidiaphragm is protected by the liver, while the left hemidiaphragm has no such protection. The left hemidiaphragm is also weaker because it has the line of embryonic fusion between the costal and lumbar components, which makes it a site of predilection [1,8]. The right-sided TDH, therefore, tends to be associated with higher energy trauma and consequently higher morbidity and mortality [5].

Missed diagnosis of TDHs can result in long-standing, chronic, hernias. These can result in the formation of adhesions between the abdominal viscera and the thoracic structures, as well as with the diaphragm itself [5]. This can lead to a more technically challenging surgery, often with poorer outcomes. Furthermore, subsequent incarceration and strangulation of the viscera can be fatal [4,5,9]. Therefore, a timely diagnosis, followed by surgical repair, is of the utmost importance. However, if the only herniated viscus is the stomach, it can be decompressed using an NG tube. This can convert the immediate nature of the surgery to an urgent one. As in our case, the patient had NG decompression of the herniated stomach at 3:00 a.m., after which he underwent surgery at 8:00 a.m. This allowed us to proceed in a more controlled manner, with dedicated laparoscopic OR staff. No adhesions were encountered, and the herniated viscera were reduced easily.

Laparotomy and thoracotomy used to be the traditional surgical options available for the management of TDHs. Since the advent of laparoscopic surgery, it has become increasingly popular to opt for a minimally invasive approach for these cases [6]. A minimally invasive approach, especially laparoscopy, is increasingly being opted for dealing with TDHs. The role of laparoscopy in the management of chronic cases of TDHs has already been explored [10]. The diagnostic and therapeutic role of laparoscopy and thoracoscopy is invaluable in these cases, where often the diagnosis is not confirmed on imaging. Trauma laparotomy can be avoided in this manner. All the advantages of minimally invasive surgery are afforded to the patient, with reduced post-operative pain and early recovery, as well as better cosmesis [2,11,12]. Another use of the diagnostic role of laparoscopy is in pregnant trauma patients who are unable to have a CT scan. Rolton et al. described the case of a lady who was 27 weeks pregnant when she suffered a high-speed road traffic accident leading to a left-sided TDH. Diagnostic laparoscopy confirmed a left-sided TDH with herniated stomach and colon and associated splenic injury. Laparoscopic repair of the hernia and splenectomy were done [13]. Kim et al. described a case of traumatic diaphragmatic rupture of the right hemidiaphragm with associated liver injury, which was successfully repaired using robotic transthoracic surgery once the patient became hemodynamically stable [3].

The usual method of management of TDHs involves the reduction of abdominal viscera into the abdomen, followed by repair of the diaphragmatic defect. This can be done via either the thoracic or the abdominal approach. Thoracic surgeons prefer the thoracic approach, whereas general surgeons usually prefer the abdominal approach. An abdominal approach is mandatory in case of suspicion of bowel ischemia and necrosis, or other traumatic injuries to the abdominal viscera.

There is some debate over the type of closure. Most defects, especially smaller ones, can be repaired primarily. The American Association for the Surgery of Trauma (AAST) classifies diaphragmatic injuries into five grades; grade I (contusion), grade II (laceration ≤2 cm), grade III (laceration 2-10 cm), grade IV (laceration ˃10 cm with tissue loss ≤25cm2), and grade V (laceration with tissue loss ˃25cm2) [14]. Larger defects (grade IV and V) usually require mesh reinforcement. The mesh can be fixed using sutures, tacks, or cyanoacrylate glue [15]. This is because, in the case of larger defects, tension-free repair requires the use of a prosthesis to bridge the loss of tissue, as well as to provide additional strength. There is considerable variation in the type of prosthesis, including simple polypropylene mesh, composite mesh, polytetrafluoroethylene (PTFE) mesh, and even biological mesh. The biological mesh is associated with a lower risk of infection, and a lower risk of displacement, as opposed to the synthetic mesh [6]. The mesh can be fixed after suturing the defect, thereby preventing protrusion of the mesh through the hernial defect. Although the mesh can be fixed on either side of the diaphragm, depending on the surgical approach used, laparoscopic placement of the mesh on the peritoneal surface of the diaphragm results in intraabdominal pressure helping to keep the mesh in place over the diaphragm [14]. In our case, we had an AAST grade III injury, and we performed primary repair of the defect, after reduction of the herniated contents, using 2/0 V-LocTM 90 sutures (Covidien Healthcare, Mansfield, Massachusetts) in a continuous manner.

## Conclusions

The conventional strategy for trauma surgery has always been an open approach. However, this is no longer the standard of care. The laparoscopic management of surgical emergencies such as acute appendicitis, acute cholecystitis, and even perforated duodenal ulcers is now standard practice. The management of delayed presentation of diaphragmatic rupture is also commonly done with laparoscopy. Now, we are moving toward a minimally invasive approach for the management of TDHs in the acute setting provided that the patient is stable, and resources are available. Furthermore, a simple step like NG tube insertion and aspiration, after ruling out fracture of the base of the skull, can be life saving in certain situations.
